# The development of a hiPSC-based platform to identify tissue-dependencies of IDH1 R132H

**DOI:** 10.1038/s41420-023-01747-w

**Published:** 2023-12-12

**Authors:** N. Z. Mehjardi, J. Kessler, A. Y. Sanin, D. Picard, P. Westhoff, Ann-Christin Nickel, C. Uhlmann, W. Shi, H. J. Steiger, M. Remke, I. Fischer, D. Vordermark, R. S. Croner, U. D. Kahlert

**Affiliations:** 1 Clinic for Neurosurgery, Medical Faculty Heinrich-Heine University and University Medical Center Düsseldorf, Düsseldorf, Germany; 2 Clinic for Radiation Therapy, Martin Luther University Halle-Wittenberg, Halle, Germany; 3 Department of Molecular and Experimental Surgery, Clinic for General, Visceral, Vascular, and Transplant Surgery, Medical Faculty and University Hospital Magdeburg, Magdeburg, Germany; 4 Department of Pediatric Oncology, Hematology and Clinical Immunology, University Hospital Düsseldorf and Medical Faculty, Heinrich Heine University Düsseldorf, Düsseldorf, Germany; 5 German Cancer Research Center (DKFZ), Heidelberg, Germany; 6 German Cancer Consortium (DKTK), partner site Essen/Düsseldorf, Düsseldorf, Germany; 7 Institute of Plant Biochemistry, Cluster of Excellence on Plant Science, Heinrich Heine University, Düsseldorf, Germany; 8 Present Address: Diaceutics PLC, Düsseldorf, Germany; 9 Present Address: Charles River, Wuppertal, Germany; 10 Present Address: Pediatric Oncology and Hematology, University Hospital Saarbrücken, Saarbrücken, Germany

**Keywords:** Induced pluripotent stem cells, Cancer models

## Abstract

The application of patient-derived (PD) in vitro tumor models represents the classical strategy for clinical translational oncology research. Using these cellular heterogeneous cultures for the isolation of cancer stem cells (CSCs), suggested to be the main driver for disease malignancy, relies on the use of surrogate biomarkers or is based on CSC-enriching culture conditions. However, the ability of those strategies to exclusively and efficiently enrich for CSC pool has been questioned. Here we present an alternative in vitro CSC model based on the oncogenic transformation of single clone-derived human induced pluripotent stem cells (hiPSC). Hotspot mutations in the DNA encoding for the R132 codon of the enzyme isocitrate dehydrogenase 1 (IDH1) and codon R175 of p53 are commonly occurring molecular features of different tumors and were selected for our transformation strategy. By choosing p53 mutant glial tumors as our model disease, we show that in vitro therapy discovery tests on IDH1-engineered synthetic CSCs (sCSCs) can identify kinases-targeting chemotherapeutics that preferentially target tumor cells expressing corresponding genetic alteration. In contrast, neural stem cells (NSCs) derived from the IDH1R132H overexpressing hiPSCs increase their resistance to the tested interventions indicating glial–to-neural tissue-dependent differences of IDH1R132H. Taken together, we provide proof for the potential of our sCSC technology as a potent addition to biomarker-driven drug development projects or studies on tumor therapy resistance. Moreover, follow-up projects such as comparing in vitro drug sensitivity profiles of hiPSC-derived tissue progenitors of different lineages, might help to understand a variety of tissue-related functions of IDH1 mutations.

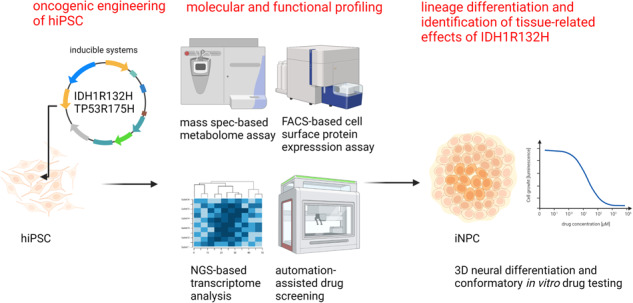

## Introduction

Isocitrate dehydrogenases (IDHs) are members of a class of rate-limiting enzymes in the tricarboxylic acid cycle involved in cellular energy metabolism, catalyzing the oxidative decarboxylation of isocitrate to α-ketoglutarate (α-KG) and CO2, and converts NAD(P)+ into NAD(P)H. Single nucleotide mutations in IDH exons are a frequent molecular parameter in a variety of cancers [1], with mutations in the R-encoding codon 132 of IDH1 representing the most frequently found alteration [2]. Mutant IDH proteins possess a neomorphic enzymatic activity, reducing α-KG) to the rare but structurally similar metabolite D-2-hydroxyglutarate (2-HG) [3]. 2-HG accumulation to millimolar concentrations in IDH-mutant cells has been shown to dysregulate epigenetic programs, such as DNA methylation, histone methylation; amino acid synthesis; hypoxic signaling or collagen maturation [4–7]. IDH1 R132 hotspot mutations are particularly frequent in leukemia [8], primary malignant brain tumors [9] and intrahepatic cholangiocarcinomas (iCCA) [10]. However, despite the above-described pan-tissue relevant features of mutant IDH, the clinical significance of R132 varies significantly between tumors of different organs. Meanwhile, the discovery of IDH1R132 has revolutionized neuropathological diagnostics [11], and the development of anti-IDH1 targeting strategies has matured into a new clinical strategy for long-term treatment success for some brain cancer patients [12], the knowledge of the biological roles of IDH1R132H, especially on possible tissue-dependent differences, is still incomplete.

The use of patient-derived (PD) in vitro tumor models represents the classical strategy for clinical translational oncology research. Using these cellular heterogeneous cultures for the isolation of cancer stem cells (CSCs), suggested to be the main driver for disease malignancy, relies on the use of surrogate biomarkers or is based on enriching culture conditions. Those strategies have previously been shown to possess certain limitations for modeling cancer stem cells. Alternative strategies to model CSC, ideally derived from a single cell of origin are needed to conduct CSC-focusing research. hiPSCs are used extensively for disease modeling purposes in various research contexts, primarily outside oncology where PD in vitro disease models are lacking [13]. Here we established and characterized a p53R175H hiPSC model with chemically inducible overexpression of IDH1R132H protein and its IDH1 wildtype (WT) paralog. We show that our approach powerfully identifies repurposes of market-approved drugs to preferentially target cells with IDH1R132H mutation as compared to those with IDH1WT, whose effectiveness can be confirmed in PD, in vitro disease models of p53 mutant IDH1R132H glioblastoma. Besides its proven usefulness in therapy treatment discovery, we believe our pluripotent platform has potential for future investigating tissue-dependent effects of IDH1R132H, especially in comparing effects on differentiation potential or drug resistance between different lineages.

## Results

### Induction of mutant IDH1 causes accumulation of intracellular levels of D2-Hydroxygluterate

**Fig. 1:**
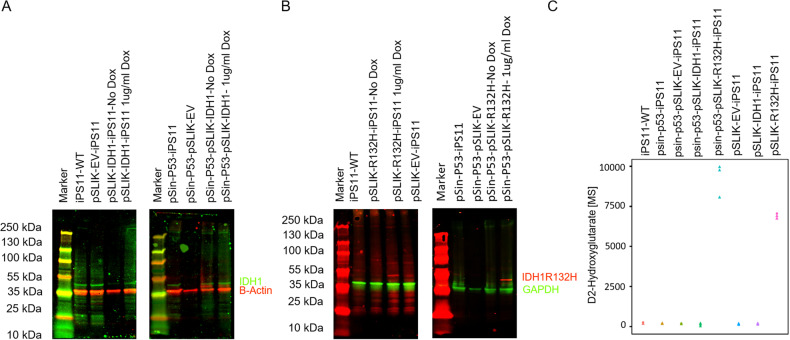
Generation of iPSC models with inducible IDH1 protein expression. Representative Western blot membranes showing protein bands of IDH1 (**A**) and IDH1R132H (**B**), IDH1 and IDH1R132H 46 kDa, GAPDH 36 kDa, beta Actin 42 kDa. IDH1R132H protein only visible after DOX induction (12 h). Quantification of D2 Hydroxyglutarate in metabolic extracts of the corresponding cells as assessed by mass spectrometry (MS) (**C**).

Protein expression of IDH1 variants was verified by Western blot after overnight treatment with Dox. Endogenous IDH1 was detected in wt and EV. IDH1 expression was observed after DOX treatment in transduced cells with pSLIK-IDH1 in pSLIK-IDH1-iPS11 and pSin-p53-pSLIK-IDH1-iPS11. IDH1- R132H protein expression was observed only in transduced hiPS11 cells with lentivirus containing pSLIK-IDH1-R132H and pSin-p53-pSLIK-IDH1-R132H after overnight induction with Dox (Fig. 1A, B). Details on the description of characteristics of our TP53 background mutation cell model, which was applied in this project, can be found in our recently published paper [14].

Since the function of IDH1R132H is defined by the generation of onco-metabolite 2HG [15], in order to verify the functionality of the described protein induction we performed targeted metabolomics to quantify 2GH in our model systems. Strikingly, models with IDH1 R132H protein are characterized by the increased level of 2-HG (after 48 hr induction with DOX). D2HG concentration was 7000 times more in pSLIK-IDH1-R132H-iPS11 and around 10,000 more in pSin-p53-pSLIK-IDH1-R132H-iPS11 (Fig. 1C). Interestingly, 2-HG accumulation is not visible after 12, increases at 24 h and highest at 48 h DOX incubation (Supplementary Fig. S1). Therefore, we define a minimum of 24 h DOX exposure is needed for the establishment of functional IDH1mut phenotype.

### Verification of stem cell characteristics

**Fig. 2:**
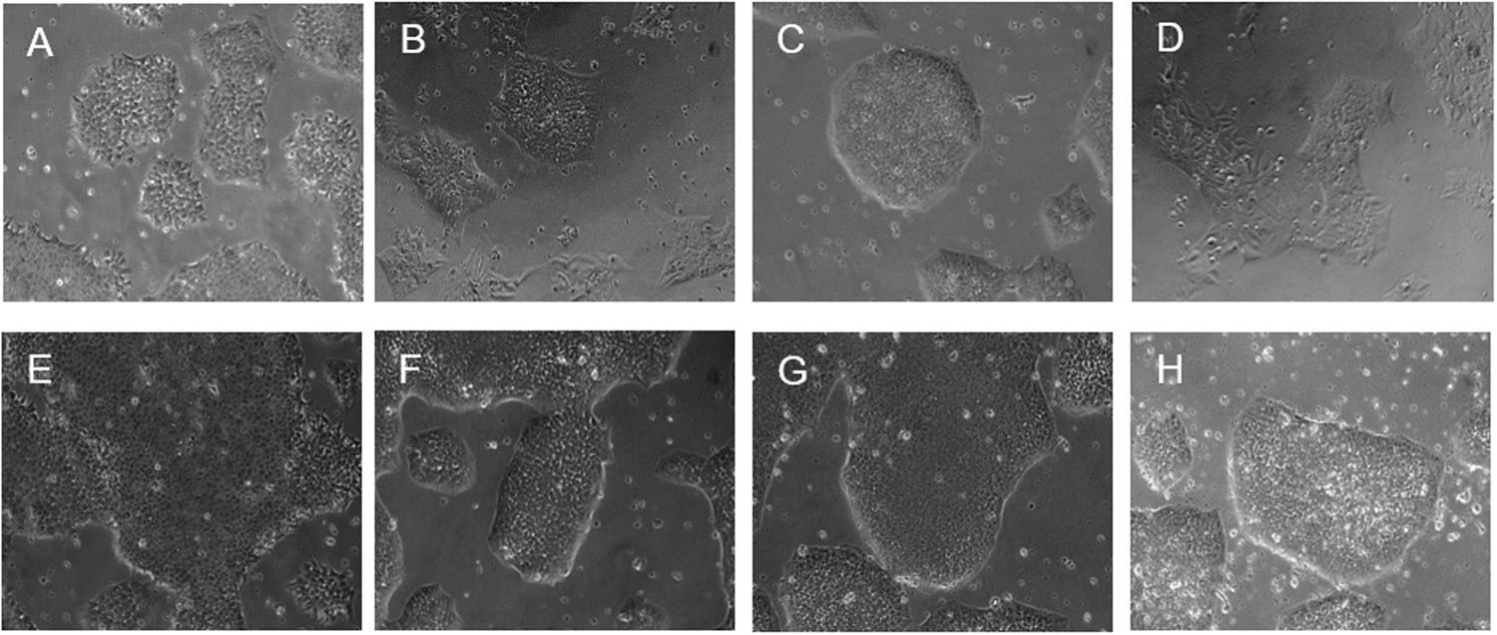
All cell models represent morphological appearance typical for pluripotent cells. **A** iPS11-wt; **B** pSLIK-IDH1-iPS11; **C** pSLIK-R132H-iPS11; **D** pSLIK-EV-iPS11; **E** pSin-p53-iPS11; **F** pSin-p53-pSLIK-IDH1-iPS11; **G**: pSin-p53-pSLIK-R132H-iPS11; **H**: pSin-p53-pSLIK-EV-iPS11.

To avoid loss of stemness because of our gene engineering, all the cell models were tested for stemness indicating features using two different assays (24–48 h DOX induction). Firstly, all cell models show typical iPSC morphology as shown by representative white filed views in Fig. 2. Secondly, flow cytometry to quantify the cell surface expression of consensus pluripotent markers (SSEA4, Nanog, Oct4 and Sox2) were executed. A summary, per cell condition, percentages of positive cells for each marker are presented in Table 1 (cells induced with DOX for one week). Our result showed that transduced cells express pluripotent markers in sufficient amounts (in average > 80% positivity) to verify the cells' stem cell condition. Representative histogram pictures of data acquisition at the FACS are shown in Supplementary Figures S2 and S3.

**Table 1:** Summary of FACS-based assessment of percentage of cell expressing pluripotency markers with IDH1-cell models tested upon induction of the transgene for 48 h.

Cell Line	SSEA4 %	hNanog %	Oct4 %	Sox2 %
pSLIK-IDH1-iPS11	98.7	50.8	82.9	30.5
pSLIK-R132H-iPS11	95.2	64.7	85.4	84.8
pSLIK-EV-iPS11	99	39.4	99.4	88.8
pSin-P53-iPS11	60.4	60.4	52.5	57.5
pSin-p53-pSLIK-IDH1-iPS11	96.7	38	80	67
pSin-p53-pSLIK-R132H-iPS11	97.5	51	84.3	91.6
pSin-p53-pSLIK-EV-iPS11	71.3	83.8	78	77.6

### IDH1R132H induction causes changes in global gene expression profile of pluripotent stem cells

**Fig. 3:**
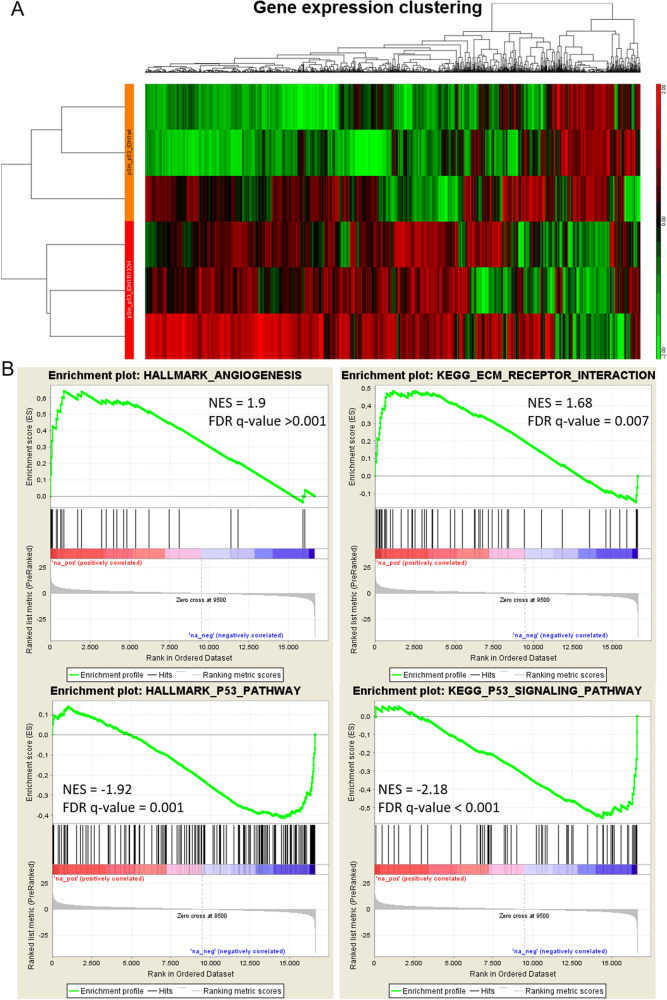
Gene expression profiling of iPS11-TP53R175H model systems. Global gene expression profiling revealed a distinct separation of transcriptome from cells with induced IDH1 and cells with induced IDH1R132H indicating the significance of this biomarker in the context of total gene expression networks in the context of human pluripotent stem cells (**A**). Gene Set Enrichment Analysis identified various pathways dysregulated in response to IDH1R132 induction such as increased expression of gene associated with angiogenesis or downregulation of p53 network indicating misbalanced DNA damage repair (**B**).

In order to identify genetic networks associated with IDH1 R132H induction, we compared the global expression profile TP53R175H - cells with induced IDH1 wild type or IDH1R132H respectively. To exclude tracing secondary effects caused by too long saturation with intracellular 2HG accumulation, we decided to conduct gene expression profiling on cells treated with DOX for 12 h. Secondly, we chose cells expressing the TP53R175H for our analysis mutant to focus on pathophysiological/clinical most relevant background based on the available models (TP53 mutation is the most frequently mutated gene in cancer). Figure 3A shows a cluster heatmap of triplications per condition (using gene ontology consensus parameters) revealing distinct differences in global gene expression patterns in response to IDH1^R132H^ induction. Moreover, confirming previous findings, our subsequent gene set enrichment analysis identified IDH1R132H induction-induced pro-angiogenic expression phenotype and suppresses p53 pathway activity (Fig. 3B).

### hiPSC expressing IDH1R132H show differences in drug resistance as compared to IDH1WT counterparts

**Fig. 4:**
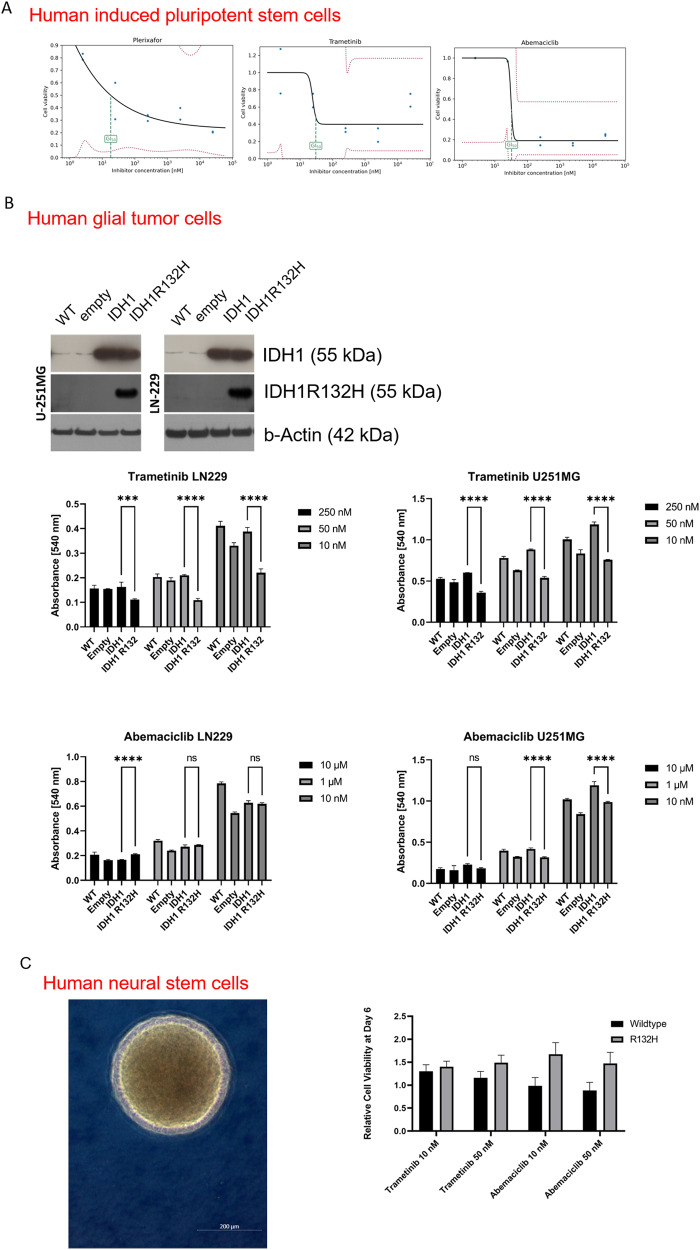
Testing effect of IDH1R132H protein overexpression on drug resistance in cells of different tissue differentiation status. Cell growth–drug response curves of iPS11_IDH1R132H on top three effective drugs out of a semi-automatic executed drug screening, as defined by dose-dependent reduction of cell growth reaching lowest 50% of growth inhibition concentration (IC50) when using minimal amount of drug (Plerixafor GI50 = 18.3 nM, Trametinib GI50 = 30,7 nM, Abemaciclib GI50 = 33,0 nM (**A**). Protein verification of IDH1 status of p53 mutant glial tumor models used in this study and results of drug testing on those glioblastoma cells. Trametinib and Abemaciclib show increased efficacy in cells expressing IDH1R132H protein as compared to their IDH1 WT counterparts (**B**). Microscopic images of human neural stem cell (NSC) and results of drug sensitivity testing of NSCs under induced transgene expression, showing a trend of increased resistance of R132H cells as to Trametinib and Abemaciclib as their IDH1 WT counterparts (**C**). ****p ≤ 0.0001 empty empty vector control, WT wildtype.

In this project, we established a technical setup allowing substance screening suitable to conduct in vitro throughput assays on hiPSC including in cells under exogenous enforced gene induction conditions. We chose the single mutation hiPSC models (TP53WT background) for this assay, as we are particularly interested in IDH1R132H-specific therapy resistance and did want to work in the genetically cleanest conditions. Figure 4A shows the drug response curves of top three performing interventions out of >170 drugs, as defined by dose-dependent reduction of cell growth reaching lowest 50% of growth inhibition (GI50) when using minimal amount of drug on IDH1R132H cells (Plerixafor GI50 = 18.3 nM, Trametinib GI50 = 30,7 nM, Abemaciclib GI50 = 33,0 nM on iPS11). A listing of 40 top-performing drugs on IDH1R132H cells and their respective IC50 can be found in Supplementary Table S1.

### Kinase inhibitors Trametinib and Abemaciclib preferentially target malignant glial tumor cells with IDH1R132H

To test the impact of IDH1R132H on drug resistance in tissue-dependent cancer context we performed in vitro sensitivity testing of engineered GBM cells with IDH1R132H to Trametinib and Abemaciclib, the two out of the top three drugs from the screening runs in the hiPSCs that showed the most clean concentration-dependent effect. Conformingly, the introduction of IDH1R132H significantly reduced the chemotherapy resistance of cells compared to their isogenic IDH1WT counterparts (Fig. 4B). The uncropped Western blots can be found in Supplementary Fig. S4.

### Neural differentiation increases therapy resistance to Trametinib and Abemaciclib

To test the impact of IDH1R132H on the functional properties in a neural context we performed in vitro sensitivity testing of NSCs to Trametinib and Abemaciclib, the two out of the top three drugs from the screening runs in the hiPSCs that showed the most clean concentration-dependent effect. To our surprise, the cell sensitivity has reverted as compared to the hiPSC data. iNSC with mutant IDH1 showed a higher resistance level than its counterparts with induced overexpression of IDH1 WT (Fig. 4C).

## Discussion

IDH1 plays fundamental roles in cellular metabolism that downstream affects a plethora of cellular and molecular programs. As a frequently occurring molecular event in leukemia, brain cancer and cholangiocarcinoma (CCA), the clinical impact of IDH1 R132 – mutation direct diagnostics or therapeutics varies largely between different tissues. As such, IDH1R132 has democratized the field of neuro-oncology whereas little impact on routine clinical approaches for patients with CCA is developed. The presented platform technology allows the functional investigation of IDH1R132H or IDH1R132H-targeting tools in a single cell of origin, isogenic controlled conditions.

With our exemplary study on chemotherapy resistance testing, we possibly identified interesting new treatment options for p53 mutant tumors glial tumors. The detected top drug suggestions are all molecular-targeted pharmaceutics approved for cancer therapy. Trametinib to block activity of signal transduction enzymes mitogen-activated extracellular signal-regulated kinases 1 and 2 (MEK1/2) and Abemaciclib is an inhibitor of cyclin-dependent kinases 4/6 (CDK4/6). Interestingly, reduced therapy resistance of IDH1R132H glial tumor cells to the two drugs was not detected in IDH1R132H U343MG cells, a cell line with p53WT DNA (data not shown, glial tumor cell line DNA mutation status see Supplementary Table S2). Clinical trial data on biliary tract cancer patients, suffering from a tumor that is frequently mutated in IDH1 locus [16], showed the inclusion of Trametinib as part of dual drug-combination treatment regime outperformed the clinical benefit of pan-FGFR kinase inhibitor BGJ398 plus IDH1Mut inhibitor Ivosidenib [17]. Trametinib may be effective against IDH1mut cells, further studies are needed. In another recent high-profile paper, it was shown that IDH1 mutation in leukemia cells caused MAPK activation, and targeting this pathway through CDK4/6 inhibitor Abemaciclib, more effectively inhibited proliferation in IDH mutant AML than in IDH wild-type AML [18, 19]. These data and our indications support CDK inhibition with Abemaciclib may be particularly effective in cancerous tissues, including cancer stem cells that carry IDH1 mutations. Of note, the current clinical trial NCT04118036 investigates the potential of Abemaciclib as part of dual-combination therapy to treat glioblastoma with no outcome data posted (www.clinicaltrials.gov, last update December 2021).

On the other hand, our comparative in vitro drug sensitivity study identifies tissue-dependency of biological functions of IDH1R132H, as neural differentiation increased the resistance of IDH1R132H hiPSC cells. This is in contrast to the results in glial or pluripotent cells. Further studies applying this model for testing genetic and cellular capacities when parallel-wise differentiating the engineered hiPSCs in different lineages may help improve our understanding of the role of IDH1 during tissue development and disease.

Our results have important relevance for the field of disease modeling. Our transcriptomic profiling clearly shows the profound effect of IDH1 induction on global gene expression household. To the best of our knowledge, this is the first evidence of the very profound impact of IDH1 R132H on human stem cell biology. Moreover, our targeted analysis on patterns of interest could replicate dysregulation in gene expression control [20–22] as well as in pathways previously already prominently associated with the biology of IDH1R132H in cancer, such as increased angiogenesis [23–25] or DNA repair [26, 27]. The strong consequences of IDH1R132H on dysregulations of the cellular epigenome are well established. Our data now extends this list to hiPSCs. Interestingly, our data indicate that the effects of IDH1 activity on DNA-methylome profile are less pronounced when acting in dysregulated p53 background, suggesting p53 mutations possibly counteract IDH1 influence on DNA-methylome regulations. Further studies, i.e. to compare activation properties of epigenetic regulating enzymes in the context of IDH1 mutant cells with and without p53 pathway dysregulation regulation are needed to verify our initial observations. Regarding the field of neurooncology, although some recent reports on establishing IDH1^MUT^ high-grade cancer cell lines from corresponding mutated patient samples emerge [28], the establishment of IDH1 mutant in vitro models usually rely on genetic engineering of cells [29, 30]. Stem cell models IDH1R132H have been reported by Rosiac-Stec and colleagues [31] as well as preliminary data on hiPSC model development published by the Klink group [32]. The availability of the latter model would be highly important for the field as it recapitulates the endogenous IDH1R132H expression rather than an overexpression of the mutant version on top of the IDH1 WT background.

We acknowledge certain additional limitations of our disease-modeling attempt. Besides the overexpression of IDH1R132H on top if IDH1 WT does not recapitulate the common mutation condition in humans, that is i.e. (mono)-allelic mutation [29], our gene engineering approach cannot control for integration side. For none of the conducted comparisons between different genetic conditions, we cannot normalize or exclude variations that may occur with lentiviral vector integration in target cells, such as integration site and quantity. Therefore, replication with different biological hiPSC generated from a different human donor, ideally increasing ethnic and gender diversity, may be relevant to confirm our observations. However, we believe our model system is a unique resource possibly supporting research and development projects in a wider area of biomedicine. The authors acknowledge that any hypothesis on the therapeutic potential of our discoveries must be confirmed with dedicated drug testing trials using patient-derived cancer stem cell disease models such as primary cancer organoids. Our efforts are in line with current global science policies and regulatory guideline developments to appreciate non-animal model systems for biomedical research, both in basic science and clinical-translational-oriented projects.

## Methods and reagents

### Human-induced pluripotent stem cell models

Human induced pluripotent stem cell (hiPSC) line from Alstem advancements (Episomal, human foreskin fibroblast; #iPS11) was cultured in 6 well plate coated with vitronectin (VTN-N) 0.5 µg/ cm^2^ in Stem Flex medium (both from Thermo Fisher Scientific). Cells were regularly passaged with 0.5 μM EDTA (Roth). Before transduction, colonies were dissociated with TrypLE^™^ (Thermo Fisher Scientific) and seeded on vitronectin. For transfection, human iPSCs were split in a ratio of 1:6 in a 6-well cell culture plate. To reach a higher virus concentration 12-well plates were used for the transduction of c-MYC and GLI1 as the transduction did not work in the 6-well plates. On the next day, the medium was changed and one aliquot of 40 μl lentivirus was added to each well. In the following 2 days, half of the medium was replaced by a fresh cell culture medium. Antibiotic selection with 1 μg/ml puromycin was started 72 h after lentiviral transduction and continued for one week. Cells were split in the selection medium if they reached a confluency of 70–80% during the selection. After the end of the selection, cells were cultivated in a selection medium containing 0.2 μg/ml puromycin.

To overexpress IDH1-Wt and IDH1-R132H plasmids pSLIK-IDH1-FLAG and pSLIK-IDH1-R132H-FLAG and pSLIK-GFP as empty vector (Addgene plasmid # 66802, # 66803 and #66844), originally generated in the Metallo lab [33] were used. These vectors contain a Tet-on system that can be activated with doxycycline exposure (Dox). Expression of the IDH1, IDH1-R132H and GFP was evaluated after overnight induction with 1 µg/ml Dox (Sigma). Moreover, we used the same IDH1 transfection campaigns in a version of previously described p53- mutation models of iPS11 [34] using TP53R175H variant (in the future abbreviated as TP53) to establish double factor models. The characterization of our generated, here used TP53 background mutation model was recently described [14]. Genetic authentication of cells was conducted as previously described [14].

### Extraction of metabolites and quantification of D2-hydroxygluterate

Forty-eight hours after Dox induction, approximately 2 × 10^6^ cells from each cell line were washed with ice-chilled isotonic saline and metabolism quenched and metabolite was extracted by adding ice-cold chloroform/methanol. Samples were frozen in liquid nitrogen for a short time and kept at −80 °C prior to gas chromatography-mass spectrometry analysis. For GC-MS analysis the samples were prepared and analyzed as described [35]. Identification of metabolites was performed with MassHunter Qualitative (v.b08.00; Agilent Technologies, Santa Clara, CA, USA) by comparing the mass spectra to the NIST14 Mass Spectral Library (https://www.nist.gov/srd/nist-standard-reference-database-1a-v14) and to a quality control sample containing 2-HG. 2-HG peaks were integrated using the 129 m/z fragment as a quantifier at a retention time 11.6 min with MassHunter Quantitative (v.b08.00; Agilent Technologies). For relative quantification, the metabolite peak area was normalized to the peak area of the internal standard ribitol.

### Flowcytometry

Stem cell marker expression of hiPSCs was evaluated using the BD Stemflow™ Human Pluripotent Stem Cell Transcription Factor Analysis Kit (Becton, Dickinson and Company #560589, CA, USA). Protocol was followed according to the manufacturers’ instructions. Centrifugation steps were extended to 10 min to increase the cell yield. Briefly, 9 wells of a 6-well plate of hiPSCs were split with TrypLE™ Select (Thermo Scientific, MO, USA) to obtain single cells (as described before). For each staining condition, single staining and triple antibody staining, 1 milion. cells were transferred to a flow cytometry tube. As a control the cells were not stained or only stained with the fixable viability dye (Becton, Dickinson and Company #FVS510, CA, USA). Afterwards, cells were washed with staining buffer (DPBS−/− + 2% heat-inactivated KnockOut™ serum replacement (KSR; #10828-010, Thermo Fisher Scientific, MA, USA)). Cells were fixed with the provided BD Cytofix fixation buffer for 20 min at RT. Cells were washed and permeabilized using the provided Perm/Wash buffer for 20 min at RT. Finally, hiPSCs were stained with the stem cell marker PerCP-Cy™ 5.5 Mouse anti-Oct3/4, PE Mouse anti-human Nanog, Alexa Fluor® 647 Mouse anti-Sox2 and the respective isotype controls for 30 min at RT, washed and measured on the CyAn Beckman Coulter (CA, USA) and analyzed with the provided software Summit V4.3.03 (Beckman Coulter, CA, USA).

### mRNA sequencing

Total RNA was extracted from snap-frozen cell pellets using the NucleoSpin RNA extraction kit (Macherey-Nagel, Germany) with DNaseI treatment according to manufacturer protocol. RNA was quantified using the Qubit RNA HS Assay (Thermo Fisher Scientific, Germany) and quality was measured by capillary electrophoresis using the Fragment Analyzer and the Total RNA Standard Sensitivity Assay (Agilent Technologies, Inc. Santa Clara, USA). The library preparation was performed according to the manufacturer’s protocol using the VAHTS™ Stranded mRNA-Seq Library Prep Kit for Illumina® V2 (Vazyme, China). Briefly, 300 ng total RNA were used for mRNA capturing, fragmentation, the synthesis of cDNA, adapter ligation and library amplification. Bead-purified libraries were normalized and finally sequenced on the HiSeq 3000 (Illumina Inc., USA) with a read setup of 1 × 150 bp. The bcl2fastq tool was used to convert the bcl files to fastq files as well for adapter trimming and demultiplexing. Data analyses on fastq files were conducted with CLC Genomics Workbench (version 10.1.1, QIAGEN, The Netherlands). The reads of all probes were adapter trimmed and quality trimmed (using the default parameters: bases below Q13 were trimmed from the end of the reads, ambiguous nucleotides maximal 2). Fastq files were imported into Partek Flow (Partek Incorporated, St. Louis, MO, USA). Quality analysis and quality control were performed on all reads to assess read quality and to determine the amount of trimming required (both ends: 13 bases 5´ and 1 base 3´). Trimmed reads were aligned against the hg38 genome using the STAR v2.4.1d aligner [36]. Unaligned reads were further processed using Bowtie 2 v2.2.5 aligner [37]. Finally, aligned reads were combined before quantifying the expression against the ENSEMBL (release 84) database using the Partek Expectation-Maximization algorithm and quantile normalized. Partek Flow default settings were used in all analyses. The data are available in data repository NCBI GEO under accession number GSE188228.

### Glial tumor models

Cell lines were retrieved from commercial source ATCC. Establishment of constructs and transduction of glioblastoma (GBM) cell lines U251MG or LN229 stably overexpressing IDH1 or IDH1R132H were performed as previously described [38]. Genetic authentication of cells was conducted as previously described [14]. GBM cells were grown in RPMI 1640 medium (Lonza) supplemented with 10% FBS (Thermo Scientific), 1% sodium pyruvate (Thermo Scientific), 185 U/mL penicillin and 185 µg/mL streptomycin (Biochrom) (cell culture medium) at 37 °C in an incubator with humidified air and 5% CO_2_. All experiments were performed with cells in the logarithmic growth phase. Cell cultures were analyzed for mycoplasma contamination at regular intervals not exceeding two weeks using the Venor®GeM Classic Mycoplasma PCR Detection Kit (Minerva Biolabs).

### Neural differentiation of human induced pluripotent stem cells

Differentiation into neural progenitors via 3D induction procedure, leading to neural stem cells (NSCs) with cellular and extracellular features found in neural tissue, was conducted similarly as previously described by us [39]. After neural differentiation, obtained free-floating suspension 3D-spheroids were cultured in poly-2-hydroxyethyl methacrylate (polyHEMA) (Sigma-Aldrich) coated T25 cell culture flasks using a neural proliferation medium. This medium consisted of serum-free Dulbecco’s modified Eagle medium and 30% F12 medium (Gibco, ThermoFisher, Germany), enriched with 2% B27 (Gibco BRL), 1% N-2 supplement (Gibco BRL), 1% Penicillin/Streptomycin (Gibco), 20 ng/ml basic Fibroblast Growth Factor (bFGF) (Peprotech, Germany) and 20 ng/ml Epidermal Growth Factor (EGF) (Peprotech, Germany). Proliferating NSCs with a diameter of 300–500 µm were cut into 100–150 µm using a tissue chopper every 8–10 days to expand the culture.

### Western blot

After running the isolated protein samples on SDS-PAGE (Biorad), they transferred to the PVDF membrane (Amersham Hyperfilm ECL 18 × 24 cm, GE). Blots were probed with antibody against IDH1-R132H (Dianova, DIA-H09, mouse), IDH1 (Cell Signaling Technologies, #3997, rabbit) and GFAP (ProteinTech Group Inc., #60004–1, mouse) or beta-Actin (Cell Signaling Technology, #4970, rabbit). The secondary antibodies goat-anti-rabbit IRDye800CW (1:10,000, LI-COR, Lincoln, NE, USA, #926-32211), goat-anti-mouse IRDye680RD (1:10,000, LI-COR #926-68070). Near-infrared (NIR) fluorescence signals were detected on Odyssey CLx Gel Imaging Scanner (LI-COR). Recently, a detailed protocol for the procedure in our lab was described elsewhere [40].

### Drug screening in human induced pluripotent stem cells

384-well plates were coated with Matrigel in mTeSR medium using our robot technology (Beckman Coulter Biomek^®^ FxP robotic workstation with attached micro-plate reader (Paradigm, now Molecular Devices, CA, USA). After coating, the plates were shortly down-centrifuged and sealed using parafilm. Single-cell suspension of the hIPSCs was prepared using StemPro Accutase Cell Dissociation Reagent (Thermo Fisher Scientific™, MA, USA) containing 10 µM Rock inhibitor (Selleck Chemical Llc., TX, Houston). In detail, the cells iPS11-IDH1R132H were washed two times with PBS followed by treatment with 1 mL accutase in the incubator with 5% CO_2_ at 37 °C for 4–5 min. The mTeSR medium was added to stop the reaction and the cells were centrifuged at 200 *g* for 5 min. The supernatant was removed and the cells were suspended in fresh mTeSR medium and counted using Trypan Blue (Thermo Fisher Scientific™, MA, USA). For the screen, 2000 cells per well were applied in 30 µL mTeSR medium plus doxycycline into a 384 well plate using Biomek^®^ FxP robotic workstation. The Next day, the cells were washed with PBS (Ca^++^ Mg^++^) (Thermo Fisher Scientific™, MA, USA) and fresh mTeSR medium was added and >130 drugs in mTeSR with 5 working concentrations ranging from 2 nM to 20 µM were applied to the cells. The cells then were incubated for 48 h, after which the readout of the cell survival was performed using the luminescence-based CellTiterGlo assay (Promega, Walldorf, Germany) according to manufacturer guidelines, except that we dilute the reaction agent 1:1 With PBS. Details about the screening method including a full list of the used drug library can be found in our previous publication [41].

For the statistical evaluation of cell growth data, to obtain drug response curves and to define drug effectivity, linear regression was used to model the relationship between growth inhibition 50% (GI50) for the fresh and cryopreserved cells, overall substances for which GI50 was reached and for which it could be numerically determined. All computations were performed in Python, Version 3.9.4. For statistical modeling, the stats models library was used [42]. Graphs were generated programmatically using the seaborn library [43].

### Validation of in vitro drug sensitivity with human glial tumor cells

The validation of the screening results in the pathophysiological context of human tumor cells was conducted by targeted approach with selected top performer substances. The sulforhodamine B (SRB) assay [44] was used to determine cell proliferation of three glioma cell lines and their genetic models on the cytotoxicity of test compounds. This method is based on the property of SRB to bind stoichiometrically to proteins under mild acidic conditions and then to be extracted under basic conditions; thus, the amount of bound dye can be used as a measure of cell mass or to measure cell proliferation. After 24 h cell settlement in standard culture conditions, the growth medium was changed and the cells were treated with different concentrations of Trametinib (10 nM, 50 nM and 250 nM) or Abemaciclib (10 µM, 1 µM and 10 nM) for 6 days. After incubation, cells were fixed with 10% trichloroacetic acid (Carl Roth GmbH, Karlsruhe, Germany) for 1 h at 4 °C. After a wash step with ice water, a solution of 0.4% sulforhodamine B (dissolved in 1% acetic acid, Sigma-Aldrich) was used for staining for 10 min. Before drying, three washing steps with 1% acetic acid (Carl Roth GmbH) were performed. After dissolving in 300 µl of a 20 mM Trizma base solution (Sigma-Aldrich) and shaking for 10 min, the absorbance was measured at 540 nm using a Tecan Spark 10 M Multimode Plate Reader (Tecan Treading AG). The dose-response curve was calculated and plotted using GraphPad PRISM (GraphPad Software, Inc., version 9, 2020).

### Validation of in vitro drug sensitivity with neural stem cells

After reaching the 8th passage, spheroids were cultured until they reached 60% confluency. Subsequently, the 3D spheres were fragmented into very small pieces by performing four successive cuts using a tissue chopper. Five chopped pieces were then seeded into each well of polyHEMA-coated 96-well plates. Following a 24-h settling period, a concentration of 1 µg/mL of Doxycycline was introduced into the wells. At 16 h post-Doxycycline treatment, Trametinib and Abemaciclib drugs were added to the wells at concentrations of 10 nM and 50 nM, while dimethyl sulfoxide (DMSO) served as the vehicle control. Cell viability was assessed at both day 0 and day 6 after drug administration using CellTiter Glo 2.0 reagent. The reagent was added in a 1:1 ratio, followed by a 15-min incubation in the dark, and the resulting luminescence was measured using a microplate reader to determine cell viability.

### Statistics

GraphPad PRISM software (GraphPad Software, Inc., version 9, 2020) with its integrated solutions for statistical evaluation was used for testing statistical significance with appropriate tests.

### Supplementary information

Supplementary Files and tables legends

Supplementary Figure S1

Supplementary Figure S2

Supplementary Figure S3

Supplementary Figure S4

Supplementary Table S1

Supplementary Table S2

## Data Availability

Cell models can be made available for academic collaboration upon reasonable request. Sequencing data is stored in NCBI Geo database under accession number GSE188228.
