# Supplementary material for: The development of a hiPSC-based platform to identify tissue-dependencies of IDH1 R132H
Source: Cell Death Discov. 2023 Dec 12;9:452. doi: 10.1038/s41420-023-01747-w (PMC10716401; doi:10.1038/s41420-023-01747-w)
Supplement: Supplementary file 1 — Supplementary Files and tables legends [file 41420_2023_1747_MOESM1_ESM.docx]

Supplementary File S1: MS-based quantification of 2HG in metabolic extracts of IDH1R132H cells induced with DOX for 12, 24 or 48 hours. Metabolite accumulation becomes visible only after 24 hours and increases further in 48 hours’ time point.

Supplementary File S2 and S3: Example histograms of FACS-based analysis of expression of pluripotency markers on the different cell models.

Supplementary Table S1: List of top 20 effective drugs out of a semi-automatic executed drug screening, as defined by cell growth reaching lowest 50% of growth inhibition (GI50) when using minimal amount of drug. A true drug effect is verified only when confirming the automatic calculated GI50 is associated to a drug concentration-deepened effect, indicated with TRUE in the columns “fit success”.

Supplementary Table S2: Verified p53 DNA mutation status of U251MG, LN229 and U343MG
