# Supplementary figures and images for: The development of a hiPSC-based platform to identify tissue-dependencies of IDH1 R132H
Source: Cell Death Discov. 2023 Dec 12;9:452. doi: 10.1038/s41420-023-01747-w (PMC10716401; doi:10.1038/s41420-023-01747-w)

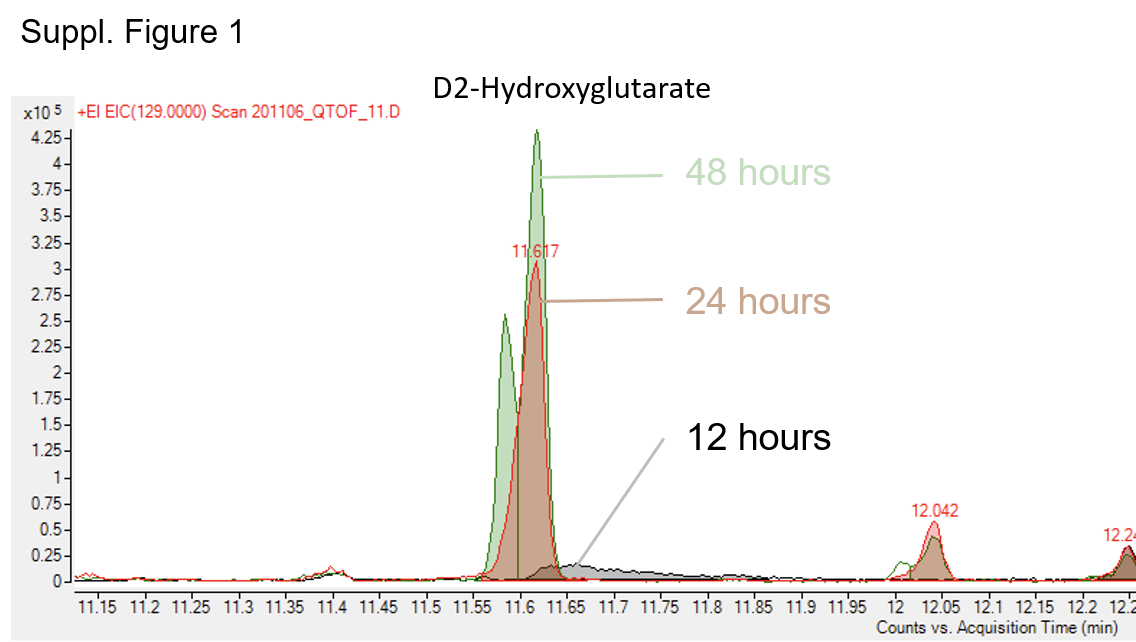

Supplement: Supplementary file 2 — Supplementary Figure S1 [file 41420_2023_1747_MOESM2_ESM.tif]

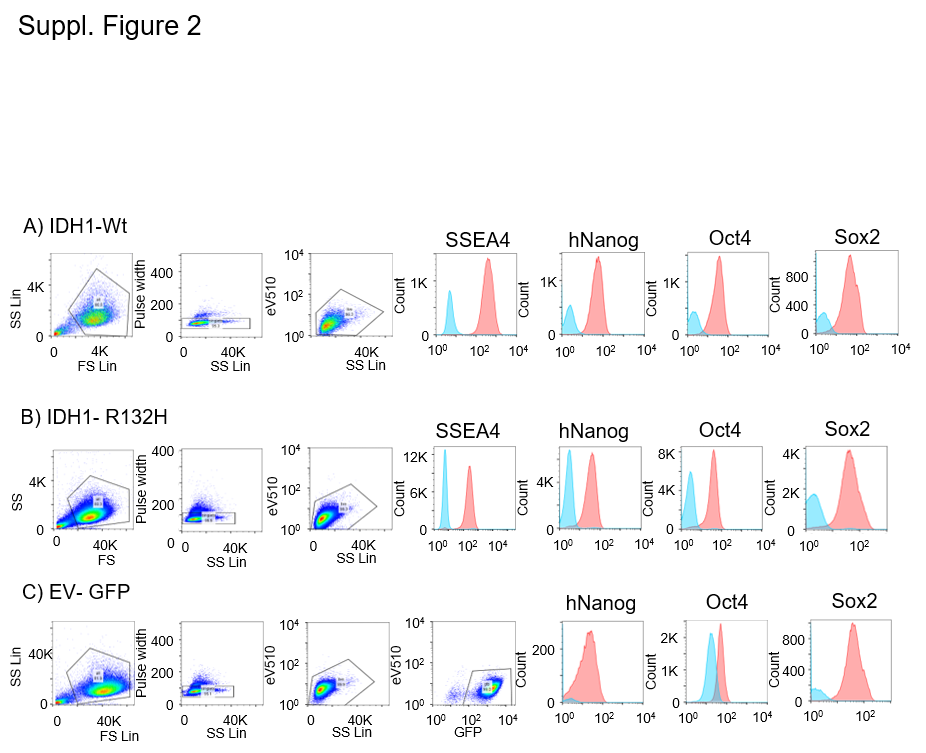

Supplement: Supplementary file 3 — Supplementary Figure S2 [file 41420_2023_1747_MOESM3_ESM.tif]

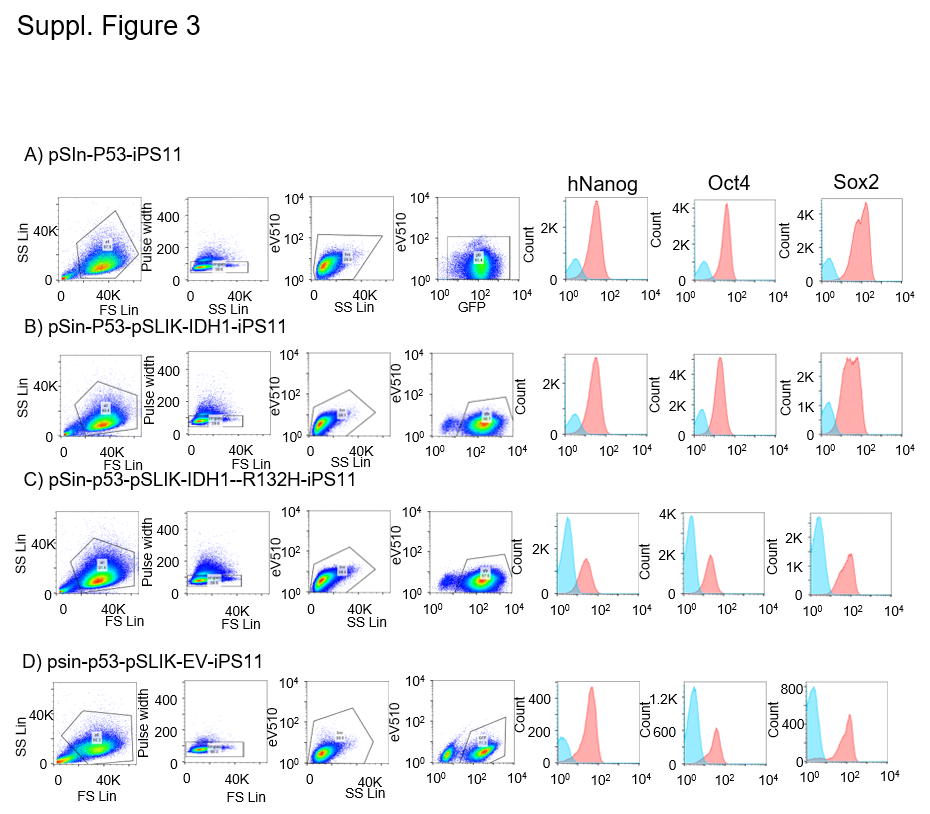

Supplement: Supplementary file 4 — Supplementary Figure S3 [file 41420_2023_1747_MOESM4_ESM.tif]

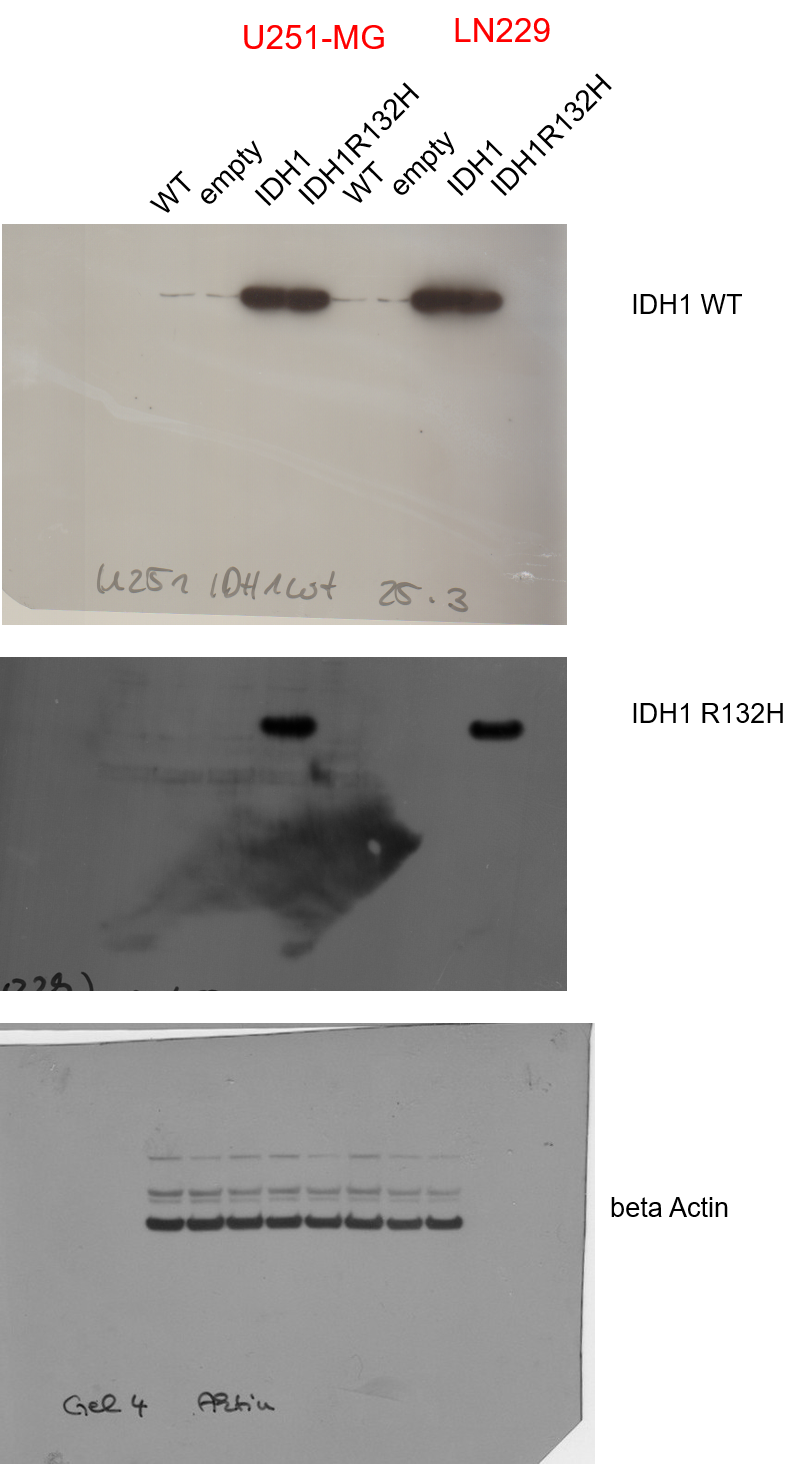

Supplement: Supplementary file 5 — Supplementary Figure S4 [file 41420_2023_1747_MOESM5_ESM.tif]

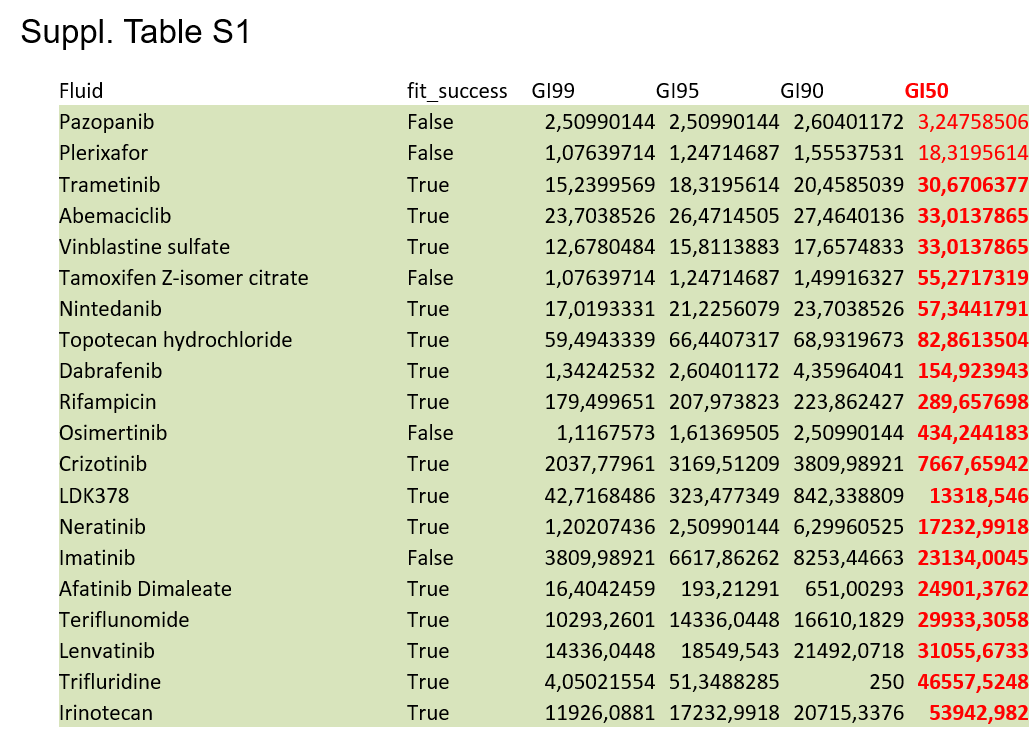

Supplement: Supplementary file 6 — Supplementary Table S1 [file 41420_2023_1747_MOESM6_ESM.tif]

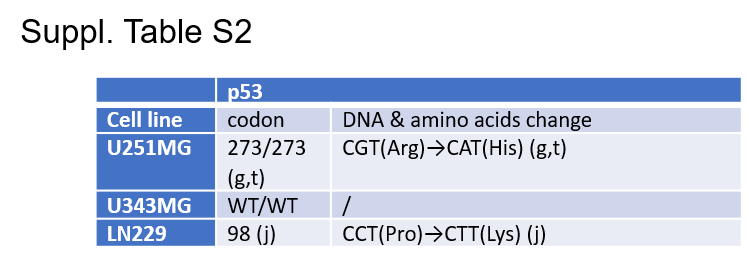

Supplement: Supplementary file 7 — Supplementary Table S2 [file 41420_2023_1747_MOESM7_ESM.tif]
